# Filters, algorithms, and the “imagination tax”: the dilemma of aesthetic self-objectification among Chinese young women on social media

**DOI:** 10.3389/fpsyg.2025.1651435

**Published:** 2025-12-18

**Authors:** Hao Sun, Ronghao Wu

**Affiliations:** School of Journalism and Communication, Qufu Normal University, Qufu, China

**Keywords:** self-objectification, body gaze, aesthetic perception, consumerism, imagination tax

## Abstract

Social media has transformed how Chinese women present themselves and provided templates for constructing self-identity; female users engage in self-representation by sharing photos, videos, and other content, and they form self-perceptions based on external feedback such as real-time interactions and virtual social engagement. This study employed semi-structured in-depth interviews with 23 Chinese female social media users aged 18-35 (20 of which were included in the final analysis), supplemented by non-participant observation across Little Red Book, Douyin, WeChat, and Weibo to examine platform recommendation logics and visual practices. Application of beautification tools and filters has increased on social media platforms, and the normalization of these technologies further alienates women's aesthetic subjectivity, rendering self-objectification a routine part of everyday practice; this imposes an “imaginary tax,” that is, the expenditure of time, money, and effort to conform to dominant beauty ideals, and factors such as occupational background and frequency of social media use play a significant role in reinforcing aesthetic objectification by deepening self-surveillance and comparison among female users. Chinese young women should therefore recognize the disciplinary logic embedded in platform algorithms and visual mechanisms, and reconstruct a healthy model of self-identification based on subjectivity and intrinsic value.

## Introduction

With the rapid development of Internet technologies, social media has become one of the primary channels for information acquisition. “While it fulfills users' needs for information, entertainment, and social interaction, it has also contributed to issues such as cognitive dissonance and social anxiety” (Jiang and Ngien, 2020, p. 155–162). Female users engage in self-presentation on social media by sharing personal photographs, videos, and textual content. In doing so, they construct an idealized self-image and obtain external feedback through the platform's interactive mechanisms. In this process, these Chinese young women often engage in the “self-gaze”, which is adopting a third-person perspective to view themselves—particularly their physical selves—and thus enacting a form of self-objectification. This paper focuses on the issues of image-related anxiety and self-worth perception among Chinese young women in the context of social media. It explores the interplay between personalized aesthetics and the “imaginary tax”, examines whether women with weaker self-awareness are more susceptible to external aesthetic objectification and investigates how platform-based interactive mechanisms contribute to this phenomenon.

## Statement of the problem

The social media environment is saturated with appearance-related content and promotions. Such content profoundly shapes women's aesthetic standards, appearance-related anxiety, and self-perception. Mainstream beauty ideals are continually reinforced through mechanisms such as group dynamics and algorithmic recommendations. As Li (2019) notes, “under specific conditions, media plays a guiding role in the construction of social gender roles” (p. 45–46). Similarly, Dong (2016) argues that under the influence of media and consumer culture, the female body gradually becomes a disciplined, symbolic and commodified body, with its subjectivity increasingly diminished (p. 56–62). Although Qu (2012) offers a diachronic analysis of Chinese women's aesthetic values and finds that, with the rise of female subjectivity, the 21st century has witnessed a trend toward aesthetic pluralism—including styles such as androgynous, sensual or intellectual beauty, (p. 121–125) the mainstream aesthetic discourse on social media platforms remains largely symbolic and standardized.

The algorithmic and personalized distribution of content has significantly accelerated the symbolic construction of aesthetic standards. Jin and Yu (2022) point out that gender representations on social media platforms are increasingly subject to a process of flattening and simplification (p. 16–22). In an examination of how collective aesthetic values are shaped, Wu (2023) argues that “women's aesthetic consciousness is largely a response to both dominant social norms and male-centered standards, thereby reflecting their gender identity” (p. 18). Social media platforms foster the homogenization of aesthetic norms through group effects and peer validation, which—under the combined pressure of consumerism and influencer culture—often generate waves of aesthetic imitation. As Tian (2023) notes, “In contemporary society, the persistent dissemination of the concept of ‘ideal beauty' by major social media platforms has had an increasingly pronounced impact, especially on university-aged youth” (p. 94–102). Specific interpretations of the female body have gradually emerged as publicly recognized standards across different platforms and online communities, thereby accelerating the proliferation of such aesthetic trends. Women's aesthetic perceptions, therefore, tend to evolve dynamically through the ongoing interaction between societal conditioning and individual self-awareness.

Capital, under the guise of seemingly free and individualized digital landscapes, silently expropriates individuals' time, reshapes their perception and strips them of agency—ultimately drawing them into vortices of desire through algorithmic control (Shi, 2024, p. 152–160). Current research on social media and women's bodily aesthetic cognition has largely focused on the platform's role as a vehicle for disseminating aesthetic values, particularly its influence on ideals for individual appearance and the diversification of aesthetic standards. These studies have underscored social media's formative power in shaping Chinese young women's aesthetic perceptions. However, insufficient attention has been paid to the complex psychological dynamics stemming from self-objectification in the context of algorithm-driven platforms and consumer culture—including how individuals negotiate between collective aesthetic pressures and internalized standards, or how to foster healthy aesthetic awareness and mitigate appearance-related anxiety. This study therefore adopted a self-objectification perspective to explore how aesthetic concepts are formed on digital platforms and how such processes influence Chinese women's self-perception.

## Research questions

Based on the above theoretical and contextual analysis, this study aims to address the following research questions:

RQ1: How do platform algorithms and filter mechanisms, through the disciplinary logic of visual culture, shape young women's self-presentation and modes of being seen on social media?
RQ2: How do young women internalize external discipline through interactive feedback on social media, leading to the psychological mechanism of aesthetic self-objectification?
RQ3: In what ways does the “imagination tax” function as a mediating mechanism between technological logic and psychological processes, reflecting the hidden costs women bear in pursuing idealized selves?
RQ4: How do young women negotiate aesthetic norms and reconstruct subjectivity amid the coexistence of algorithmic discipline and cultural pressure?

## The digital gaze: the bidirectional construction of female aesthetic perception and self-objectification

### The mediating effect of self-objectification on social media

The theory of self-objectification was first proposed by Fredrickson and Roberts (1997) and refers to the act of viewing oneself—particularly one's physical self—from a third-person perspective. According to this view, women in an appearance-centered society are frequently subjected to external sexualized gazes, which leads them to perceive themselves as “objects” in the eyes of others, thereby engaging in constant self-surveillance and evaluation of their appearance. This theory encompasses two key concepts: objectification and body monitoring. Objectification refers to the internalization of an external, gendered gaze in which women come to view themselves as “observable objects” and base their self-worth primarily on physical appearance and bodily presentation. Body monitoring involves the continuous surveillance and evaluation of one's appearance in an effort to conform to external beauty standards and obtain social approval.

Self-objectification thus represents a psychological state in which external values are internalized; it may be chronically present or triggered by specific situations. Depending on situational factors, self-objectification can be categorized into two types (Noll and Fredrickson, 1998): state and trait self-objectification. State self-objectification refers to a context-dependent response that varies according to the specific triggering situation. In contrast, trait self-objectification is a stable internal disposition that persists regardless of external context; it reflects the degree to which an individual routinely adopts an observer's perspective on her body in daily life. Self-objectification has been significantly amplified on social media platforms and demonstrates a pronounced mediating effect as women increasingly focus on appearance-centered expressions, which in turn influence their behaviors, perceptions of appearance and identity, as well as overall mental health.

#### The amplifying role of self-objectification

Social media reinforces beauty standards and gender roles. It thus functions as a major driving force behind self-objectification. Images and videos shared by users often contain extensive bodily information that emphasize appearance and physical evaluation. In participating in such activities, female users are prompted to engage in more frequent self-monitoring and modification of their appearance, thus further reinforcing their objectified self-perception.

#### The mediating effect of social comparison and feedback on body image

The aesthetic pressure imposed by social media increases women's anxiety about their physical appearance. When women are constantly exposed to digitally edited and idealized images of others, self-objectification drives them to evaluate their own appearance against these unrealistic standards, often resulting in dissatisfaction. According to social comparison theory, in the absence of objective standards, individuals evaluate themselves by comparing with others (Festinger, 1954). Self-objectification leads Chinese young women to compare themselves with idealized representations, resulting in feelings of inferiority and anxiety. Empirical studies suggest that appearance-based comparisons on social networking sites induce perceived peer appearance pressure, which in turn indirectly leads to body dissatisfaction (Liang et al., 2022, p. 444–448). The effect of online sexual objectification experiences on female college students' appearance anxiety: The mediating role of body image comparison and the moderating role of self-objectification. Women's cognitive perception of their bodies affects their emotional experience and further influences behavioral regulation.

#### The cyclical construction of self-perception and aesthetic feedback

Self-objectification in the context of social media exerts a negative impact on mental health through mechanisms such as appearance anxiety and social comparison. As the degree of self-objectification increases, individuals bear a heavier psychological burden, and social media thus becomes a latent driver in both the dissemination of aesthetic standards and the emergence of psychological distress. The mediating role of self-objectification is not unidirectional; rather, it is part of a cyclical feedback process. Women internalize external aesthetic standards and, in striving to meet these ideals, intensify self-presentation and social comparison. The feedback mechanisms embedded within social media platforms further reinforce such behaviors, thus creating a perpetuating cycle of objectifying gaze. This feedback deepens the degree of self-objectification and exacerbates its negative influence on mental health.

### The role of aesthetic cognition in shaping women's media images

Within the visual cultural field dominated by consumerism, aesthetic standards have become increasingly symbolized through repeated circulation and are eventually internalized as socially accepted norms. These norms subtly influence the construction of the female image in the public consciousness. The voices of Chinese female users on social media are articulated through visual outputs, textual narratives and cultural products that challenge traditional aesthetic paradigms and attempt to broaden the boundaries of female representation through diverse aesthetic expressions.

#### Media-driven image exhibitionism

The dissemination of media creates a self-reinforcing aesthetic system. Female images presented by the media are often prototypical representations of prevailing aesthetic norms and, through rapid circulation across digital platforms, significantly extend their influence. This diffusion gives rise to a dominant system of visual culture online that shapes both individual aesthetic cognition and broader cultural constructions. However, algorithmic designs—predominantly developed by male programmers—cannot fully eliminate the subjective inclinations of their creators. As a result, the content delivered tends to reflect gendered preferences, subtly constraining female users' interests and behaviors. Women are thus often guided into a framework of what is deemed “appropriate for them” that overlooks alternative, yet equally valid, possibilities.

#### Technologically empowered aesthetic standardization

Foucault proposed the theory of the “disciplinary power of the body” and argued that, in every society, the human body is subject to the power of control. Through the power of beauty filters and algorithms, women's bodies are subjected to increasingly refined management and regulation, that has resulted in a trend of aesthetic homogenization. Aesthetic ideals empowered by technology are increasingly solidified into new “beauty” metrics through the high-frequency repetition of sharing on social media.

For example, on platforms such as Douyinor Little Red Book, users often unconsciously activate beauty filters when using the apps to blur facial flaws, enlarge their eyes, adjust the shape of their face and constantly retouch their appearance. Meanwhile, social media algorithms tend to prioritize aesthetically pleasing and high-quality content, thus accelerating the dissemination of filtered aesthetic images. Although beauty enhancement technology does, to some extent, enhance user engagement, this standardization of aesthetics exacerbates appearance anxiety and gradually leads individuals' aesthetic preferences to converge under algorithmic guidance.

#### Symbolization of the body in society

Capital selectively extracts exemplary bodies from the masses and offers the public a referential standard of bodily aesthetics to lead aesthetic trends and induce bodily consumption (Du, 2022, p. 124–125). With the rise of consumerism, female images have gradually become part of the logic of commodification and have been packaged as symbols closely associated with success, happiness and social status. The aestheticization of everyday life often implies the consumption of aesthetic meanings attached to commodities, which is a key component of symbolic consumption (Peng, 2022, p. 129–137).

In this process, consumers seek to achieve the ideal aesthetic goals presented by media through the consumption of specific products, thus revealing the underlying symbolization and commodification of female imagery: The female aesthetic image has been reduced to a visual icon. For instance, women appearing on Douyin often possess standardized facial proportions, such as large eyes, a high and straight nasal bridge, delicate nose tips and relatively plump lips. As Baudrillard argues, consumption is not about the possession of material goods, but rather about the imagination of identity. Young women thus reconstruct and identify with their own body images through acts of consumption (Li and Li, 2022, p. 78–85).

### “Platform taming” and the “gaze of the other”: the feedback presentation of female subjectivity after disciplinary internalization

Different social media platforms play distinct roles in the dissemination and construction of aesthetic values. Take Douyin, for example: the platform rapidly disseminates content with iconic aesthetic styles through short videos, such as fashion trends labeled as “mélange aesthetic” or “dopamine dressing”, as well as symbolic beauty ideals like “pale, thin and young”; “oval-shaped face”; or “A4 waist”. Through the promotion of “filter culture” and “perfect body” ideals, social media has constructed a highly curated system of self-presentation that reinforces users' awareness of bodily optimization during self-display.

#### “Platform taming”: algorithm-driven aesthetic internalization

Social media platforms use algorithmic recommendation systems to deliver highly tailored content. Through feedback mechanisms such as likes and views, users gradually align with dominant aesthetic norms. These algorithmic recommendation mechanisms often reinforce specific types of content, thus influencing users' self-presentation strategies. Impression management on social media is not only an active behavior initiated by users but is also shaped by algorithmic curation. When a photo that conforms to mainstream beauty standards receives a large number of likes and positive comments, the poster not only gains instant emotional gratification but also a reinforced identification with that beauty standard. This mechanism operates repeatedly and gradually consolidates users' definition of “beauty” and internalizes it as their own aesthetic norm. This process may be conceptualized as “platform taming”, as the platform filters and recommends content in a manner that domesticates users' behaviors and modes of self-presentation to align with the algorithmic logic and dominant values. Through continuous interaction, female users' aesthetic standards increasingly conform to platform-imposed templates, while their personal style and individual expression are gradually diminished and replaced by externally disciplined forms of self-expression.

#### “The male gaze”: the influence of external feedback on women's self-perception

Women's representations on social media platforms often exist under the condition of being constantly subjected to “the gaze of the other”. As Peng (2022) notes, whether individuals alter their appearances through cosmetic or technological means, the criteria and direction for evaluating and modifying their looks remain predominantly shaped by the perspectives of others (p. 129–173). Social interactions have made this gaze increasingly frequent. Shaped by external evaluation mechanisms such as likes and comments, women's images are not merely outcomes of self-expression but also responses to social expectations and aesthetic norms. Social media platforms reinforce this “gaze of the other” by algorithmically promoting symbolic aesthetics. When women post content that aligns with dominant aesthetic values and receive approval, they are motivated to convert this external validation into a personal incentive.

This external feedback encourages the internalization of others' aesthetic expectations, which results in the disciplining of the self and ultimately transformation of external standards into self-identification. As this process is repeated, women may enter a persistent state of self-monitoring and often becoming overly dependent on a specific image. On platforms such as Douyin and Little Red Book, women tend to adopt mainstream visual representations to gain more recognition, often overlooking their authentic inner feelings in response to the external “gaze”. Ultimately, women's subjectivity is imperceptibly shaped and disciplined by the aesthetic norms of the platform.

#### Feedback representation of female subjectivity

The concept of the “imagination tax” refers to the cost individuals incur in altering their external appearance to meet societal expectations. Social media enables everyone to showcase themselves, thus participating in the accumulation of social capital. However, this act of self-presentation comes at a cost. Individuals often feel compelled to conform to the prevailing beauty standards that are popular on social media platforms and may even fall into a “beauty trap”.

Against this backdrop, the “imagination tax” manifests in the form of the time, energy and money spent on constructing and embellishing one's image to project an idealized self to others—a process that entails self-internalization. However, this internalization is not merely a one-dimensional, passive acceptance. Some female users, upon becoming increasingly aware of this disciplinary mechanism, begin to reshape their self-image through reflection and resistance. That is, they actively resist the monolithic aesthetic standards imposed by the platforms and attempt to redefine their self-image. This involves both the acceptance and internalization of external discipline, as well as the awakening and articulation of self-subjectivity.

## Methods

This study employed in-depth interviews as its primary research method. From July to December 2024, young Chinese female users aged 18 to 35 who were active on social media platforms such as Little Red Book and Weibo were targeted through a combination of purposive and snowball sampling (see Table 1 for Respondent Characteristics). This resulted in semi-structured, in-depth interviews with 23 participants, primarily conducted online via Tencent Meeting. All interviews were audio-recorded, transcribed, and anonymized, and 20 interviews deemed most representative were selected for detailed analysis.

**Table 1:** Respondent characteristics.

**Age (years)**	**Percentage (%)**	**Social media usage frequency (exceeds 5 hours daily)**	**Percentage (%)**	**Education level**	**Percentage (%)**
18–24	65.0	High-frequency usage	30.0	Junior College	20.0
25–35	35.0	Normal usage	70.0	Undergraduate	50.0
				Postgraduate and above	30.0
Most female university students surveyed were from humanities and social sciences, followed by medical fields, with the fewest from STEM disciplines.				**Primary platforms used percentage (%)**	
**Occupation**	**Number (individuals)**	Related occupations: student; advertising professional; new media professional; external government staff; Unemployed; Design institute staff		Little red book	90.0
				Douyin	40.0
Student	13			WeChat Moments	60.0
Non-student	7			Web	30.0
24.20,15.3**Self-objectification cognition classification (self-rated)**		**Percentage (%)**	**Aesthetic trend perception**		**Percentage (%)**
High aesthetic influence		25.0	Diversification		40.0
Moderate aesthetic influence		25.0	Conventionalization		30.0
Low aesthetic influence		50.0	Homogenization		30.0

Given that the study focused on the aesthetic practices of young women on social media, university students, as active participants in online culture, were particularly informative: their modes of expression and aesthetic preferences on digital platforms clearly reflect the deeper characteristics of contemporary media culture. Compared with other groups, university students' aesthetic perceptions are more susceptible to algorithmic mechanisms and media content, producing more salient feedback and better illustrating aesthetic changes in a society shaped by constant visibility. Although university students accounted for a majority of the sample, women of different professional and educational backgrounds were also included to enhance the diversity and comparative potential of the sample. In terms of disciplinary backgrounds, most participants were majoring in the humanities and social sciences, followed by medicine, with fewer from science and engineering, reflecting certain differences in disciplinary thinking. The social media usage habits and aesthetic perceptions of these female users constituted the core focus of this study. In addition, non-participatory observation was conducted across Little Red Book, Douyin, WeChat, and Weibo, with particular attention to algorithmic recommendation logics, user interaction patterns, and popular content types, thereby capturing the diverse interests of female users across social media platforms (see Table 2 for the Interview outline).

**Table 2:** Interview outline.

**Interview theme**	**Interview content (partial)**
Basic information	Age, occupation, education level
Social media usage habits	1. Which social media platforms do you typically use, and what activities do you usually engage in on these platforms? 2. Approximately how much time do you spend on social media each day? 3. When posting or browsing social media, how do likes, comments, or follower counts influence your behavior, emotions, or self-perception? Could you provide an example? 4. After posting content, do you consider or adjust your future posts based on feedback? What factors mainly influence these adjustments?
Social media and appearance anxiety	1. In what ways do you pay attention to your appearance or self-image while using social media? 2. Have you ever encountered social media content that made you feel differently about your body or appearance? Could you share an example? 3. When posting such content, do you consider others' evaluations or feedback on your appearance? How does this consideration influence your attention to and adjustment of your appearance or self-image?
Objectification cognition and self-expression	1. What type of content do you usually post on social media? Could you give an example? 2. How have your interactions on social media influenced your understanding of your appearance and self-worth? 3. How do you perceive the representation of women on social media? What role do you think audience comments or evaluations play in this representation? 4. What do you think will be the future trends in how social media shapes women's aesthetic perceptions and self-cognition? 5. To better understand your self-perception, please rate your level of satisfaction with your “digital self” on social media and your “real self” in everyday life, on a scale from 1 to 10 (10 indicating very satisfied). Please also briefly explain the main reasons that influenced your ratings.

After organizing the interview transcripts, we analyzed the extent to which the participants' aesthetic perceptions were influenced by social media. A more comprehensive understanding of the social media usage patterns of these Chinese young female users was constructed by combining participants' subjective accounts with participant observations conducted within the social media platforms. Based on the participants' general usage of social media and their self-reported cognitive responses during engagement, the interviewees were categorized into three groups: “deeply affected by aesthetic perceptions”, “moderately affected” and “slightly affected.”

Building on this, to gain a more comprehensive understanding of participants' self-perceptions, the study included a questionnaire on satisfaction with the “digital self” and the “real self.” Participants rated their “digital self” on social media and their “real self” in everyday life on a scale from 1 to 10 (with 10 indicating complete satisfaction) and briefly explained the main factors influencing their ratings. This approach provided qualitative evidence for analyzing the variation in the extent to which participants' aesthetic perceptions on social media were affected, as well as the underlying psychological mechanisms. Representative quotations from interviewees are listed in Table 3 Interview records and analysis.

**Table 3:** Interview records and analysis.

**Theme**	**Sub-theme**	**Representative original statements from interviews**
Platform positioning shapes user intentions and reasons for use	Differentiated platform purposes	Participant 3 (Dong Yan): Primarily uses Little Red Book and WeChat—for killing time or entertainment and searching for solutions to daily problems. WeChat is more for life, while Little Red Book is for learning and sharing practical content.
	Differences in social interaction	Participant 7 (SZY): I don't really like most social media platforms. I only use WeChat to receive notifications. I mainly interact with others on Little Red Book, QQ, and Douban, and on Douban, I collaborate with friends to create all-female team games.
	Differences in information acquisition	Participant 2 (Immortal):I use Weibo to follow the news, Douyin for entertainment and sharing, and Little Red Book like Baidu. Whenever I need information for learning or daily life, I almost always search on Little Red Book.
Social media influences users' aesthetic perceptions	Social comparison and body differences	Participant 5 (Yoyo): Back in high school, photo-editing apps with stickers were popular, and I used them because my classmates did. Later, I saw a classmate start working out in college and go from chubby to slim, which made me want to lose weight too. At that time, it was mostly blind conformity. Now, the influence is weaker; I feel only slight envy and am more affected by the people around me than by internal comparison.
	Internalized aesthetics and algorithmic reinforcement	Participant 8 (C): Social media mainly influenced my makeup choices, while my body shape was less affected as long as I'm healthy. I used to think online makeup trends were too heavy, but after seeing them frequently, I started to accept and even appreciate them. My aesthetic perception has gradually changed.
	Aesthetic imitation and recognition cues	Participant 9 (Cheri fra):Social media has indeed influenced my aesthetic. On Little Red Book, I tend to prefer round or square faces, elegant or Hong Kong-style makeup. When I see something I like, I try to imitate it. I didn't think this way before, but after browsing, I started to feel it's beautiful and a bit ‘platform-driven', gradually forming my own perception of beauty.
	Style variation and self-consistency	Participant 18 (Xiayuze): Overall, I don't think my aesthetic perception is influenced. I do follow popular trends to some extent, but my personal style remains unchanged.
Professional background shapes users' aesthetic orientations	Media literacy and defensive strategies	Participant 15 (Sweet Tiantian): When I browse Little Red Book, I tend to remain neutral and critical in my thinking. I don't get emotionally swayed. Although the titles or images may be eye-catching, I can usually tell right away if the content is promotional or advertising in nature.
	Professional ethics and resistance	Participant 3 (Dong Yanz): When I see short videos about appearance, makeup, or weight loss, I am not influenced. Because I work in advertising, I am sensitive to this kind of content, so I do not get swayed easily.
		Participant 10 (Su Xingxing): I study in a medical-related field, so I don't support women pursuing extreme thinness. For example, someone 1.7 meters tall but under 100 pounds is unhealthy. Excessive dieting can harm the body, and I just don't understand such pursuits.
	Technical awareness and rational thinking	Participant 17 (Liu Yuhan): Because my academic background involves programming and internet technologies, I understand how software is developed. What's called “personalized recommendation” or “big data targeting your preferences” is, in essence, just lines of code that filter information for you. From a technical perspective, there's nothing inherently “human” about it—what you see is the result of programmers stacking code.
Platform algorithms contribute to the objectification of women	Comment systems and self-reflection	Participant 9 (Cheri fra): Sometimes, when I see posts on Xiaohongshu with many comments, I start to wonder if I'm similar to the original poster or to someone mentioned in the comments. I can't help but take on those perspectives when viewing myself.
	Self-evaluation and internalized perception	Participant 16 (Jiao Yidian): At first, I didn't care much about facial flaws. But when I started learning makeup from beauty bloggers, I realized I looked different from them. For example, drawing eye bags looked awkward on me, and I unconsciously magnified my own imperfections.
	Social evaluation and gendered gaze	Participant 11 (Zhao Xinzhe): On social media, female appearances are more frequently judged than male ones. As long as men aren't unattractive, they usually receive positive comments; but even beautiful women often face harsh or hostile criticism.
Users express expectations for personalized and diversified aesthetics	Fluidity and re-standardization of aesthetics	Participant 13 (Maomao): On social media, users who scroll passively tend to see more conservative content. But those who consciously pursue bodily or spiritual freedom actively seek diverse aesthetic styles. Social media itself is becoming more personalized.
		Participant 14 (Pufflover): Trends today are becoming more diverse. Whether in terms of body shape or appearance, I feel like society is gradually moving away from the old beauty standard of being pale, thin and young. Recently, many platforms have criticized that ideal, and I think that's a positive shift.
	Aesthetic subjectivity and resistance to normalization	Participant 10 (Su Xingxing): It feels like a contest between different standards. Various groups of women advocate for different aesthetics, and after these collisions, a mainstream standard emerges—diverse at first, but eventually unified and popularized.
		Participant 20 (Yu Haiyue): I think when a video gets a lot of likes, the platform continues to push it to more users. The aesthetic behind that kind of content tends to remain fixed—it doesn't really change.
	Personal awareness can counter and reshape the aesthetic trends set by the platform	Participant 19 (Collins): I enjoy trying out different styles. My skin is darker, and certain clothes can make it appear even darker. But if I view dark skin as a problem, then I'm already being disciplined by a particular aesthetic standard. In fact, by experimenting with different looks, I've expanded that standard. It's the standard itself that tries to turn me into its slave.

Based on the observations of their behavior, the users' habits, social preferences, platform content and modes of interaction all appeared to influence their aesthetic perceptions and consumption behaviors. Social media's algorithm-driven recommendation systems, along with seamless content transitions and instant interaction mechanisms, draw users into a “screen-swiping” aesthetic ecology, often without conscious awareness. Under such fragmented usage patterns, dominant aesthetic values may infiltrate users' everyday aesthetic preferences in subtle and implicit ways. We thus analyzed the data in light of the following question: How do prevailing aesthetic trends penetrate users from diverse backgrounds?

### Results: the self-objectification within the “gaze” of social media

An analysis of the interview data revealed that the process of aesthetic self-objectification among Chinese young women on social media is shaped by multiple interacting factors, including algorithmic recommendation systems, frequency of platform use, occupational background and interaction mechanisms. These elements collectively contribute to the transformation of aesthetic cognition.

#### Disciplinary factors within the platform

Different algorithmic mechanisms result in varying operational characteristics, user demographics and content styles across platforms. Users tend to engage with social media platforms with different intentions and objectives. For instance, on the Little Red Book platform, users' emotional experiences are influenced and even produced by algorithms, and this technologically mediated emotional production encourages users' sustained participation on the platform (Bie and Zeng, 2024, p. 15–23). Douyin disseminates trend-related content—such as makeup, fashion and fitness—rapidly through short videos, and due to its fast-paced updates and short content cycles, it generates highly immediate aesthetic trends. Chinese Young female users can quickly access fashion trends while scrolling through videos, and they are stimulated by visual and auditory effects, which provoke a desire for imitation. Although users' purposes, social interactions, and information orientations differ across various platforms, their aesthetic perceptions within algorithmic environments are easily “guided.” Decisions such as “what to wear” and “what style to choose” are no longer made actively but instead are passively received. This phenomenon reveals the disciplinary factors embedded in platforms: algorithms continuously reinforce established aesthetic norms through content recommendation, interactive mechanisms, and community feedback, leading users to unconsciously internalize the aesthetic standards defined by the platform and further anchoring external evaluation as a key reference in constructing self-value.

#### Tamed aesthetic standards

High-frequency use of social media may trigger “social comparison”, which in turn affects individuals' evaluations of their own aesthetics and affects their psychological well-being. Social comparison theory, first proposed by the social psychologist Festinger (1954), posits that individuals possess a fundamental motivation to evaluate their own opinions and abilities, and in the absence of objective standards, they tend to evaluate themselves by comparison with others. Content on life-sharing social media platforms is often enhanced through technical means such as filters and beautification features. Users are frequently exposed to representations of a “perfect” life—such as beautiful travel photos, stylish fashion looks or successful fitness transformations—and tend to engage in upward comparisons with those who appear more successful, physically attractive or glamorous than themselves. While such comparisons may motivate individuals to pursue improvement, they often also trigger negative emotions such as anxiety and dissatisfaction. When users observe the “idealized” states presented by top influencers, it may prompt self-scrutiny. Frequent exposure to such content may lead respondents to feel dissatisfied with their own appearance or lifestyle. They may come to believe that they fall short of the prevailing standards, thereby developing aesthetic anxiety. One interviewee, Yoyo (24 years old, currently a second-year PhD student), stated:

Back in high school, many photo editing apps were very popular—like the ones where you could add a little bear sticker or decorative labels. I would ask my classmates about them and follow along to use the same apps. That was one aspect. […] Another instance was when I suddenly noticed that a high school classmate had started working out in college—she used to be chubby, but then became very slim. […] I thought to myself, “Oh my god, even she's making an effort now, I should be doing something too—I need to start losing weight.” It was a kind of blind conformity. […] But now, it doesn't affect me as much as it did back then. At most, I might feel a little envy. […] As to whether this stems from internal self-comparison or from others' evaluations, I'd say it mostly comes from comparing myself with people around me. (Interview, Yoyo)

As users evaluate whether they are “beautiful enough” or “successful enough” within virtual networks, they may experience self-doubt, diminished self-confidence and even real-life consequences such as social withdrawal or psychological issues. Content on social media is often highly curated and beautified, which makes it difficult for ordinary individuals to replicate. This disparity can cause users to perceive their real lives as inadequate, thereby intensifying anxiety and psychological pressure. As users become entangled in comparisons between the virtual and the real, they may devote increasing amounts of time to seeking validation, which further reinforces unrealistic aesthetic standards and leads to a decline in mental health.

#### Aesthetic flexibility from a professional perspective

The constraints on bodily control are omnipresent under the ideological veil of beauty capital. Chinese young women, through habitual engagement with media, gradually internalize platform-based control as self-discipline, which gives rise to negative bodily imagery Liu, (2023), pp.103-110. Professional background appeared to influence both the aesthetic preferences of interviewees and their purpose in using social media to a certain extent. We found that women engaged in professions related to algorithmic logic and social media information were more inclined to construct personal aesthetics actively, whereas others tended to accept external influences passively.

Interviewee Liu Yuhan (22, a student majoring in computational mathematics and applied software) stated:

Because my major involves frequent interaction with web programming, I understand how software is developed. Whether it's so-called personalized recommendations or big data identifying your preferences, it all comes down to lines of code that help filter various types of information. From a professional perspective, there's really nothing inherently “human” about it—it's all just the outcome of code written by developers. Speaking for myself, I went through a transition—from caring a lot, to caring less, to now barely caring at all. So yes, my academic background probably played a role in that process. (Interview, Liu Yuhan)

Interviewee Dong Yan (30, advertising professional) stated that due to her work in the advertising industry, she had developed a heightened sensitivity to information on social media. She described herself as having become somewhat “desensitized” regarding short videos and content related to appearance, beauty or weight loss. Her use of the term “desensitized” reflects her ability to critically recognize and filter out content she perceives as overly commercialized or pandering, as well as to resist being easily swayed by such externally imposed aesthetic values.

#### “Cost burden in platform interactions”

The interactive mechanisms of social media platforms may inadvertently accelerate the objectification of female identity, prompting young Chinese women to increasingly construct their self-worth based on external evaluations. This interaction model encourages women to post content likely to attract attention, which is often centered on physical appearance, thereby reinforcing reliance on external validation as the main measure of self-worth. Over time, this may generate psychological, temporal, and consumption-related burdens—what can be described as digital liabilities.

Interviewee Zhao Xinzhe (27, industrial designer) remarked:

“I think that on social media, women's appearances are evaluated more frequently than men's. You'll notice that as long as a man is reasonably attractive, most comments tend to be positive. But for women, even if they are very beautiful, there are still discordant remarks. Even when a woman's appearance is flawless, other details will be attacked. Sometimes these evaluations have nothing to do with appearance at all.” (Interview, Zhao Xinzhe)

Such pronounced gendered differences make female users more attentive to how others evaluate their appearance, often at the expense of expressing their individuality, intellect, and intrinsic value. This deepens objectification, normalizes aesthetic shame, and imposes clear psychological costs.

Interviewee Cheri fra (20, graduate student) added:

“Sometimes, when I see someone post a photo on Little Red Book and there are many comments underneath, I find myself thinking: am I like the original poster?” (Interview, Cheri fra)

This passive identification and projection of others' evaluations exemplify the psychological dimension of aesthetic objectification. As reliance on prevailing trends intensifies, young women may continuously adjust their appearance, neglecting personal traits and intrinsic value. Hashtags and trending topics on social media carry gendered stereotypes; topics often contain explicitly gendered attributes such as “sexy”, “cute”, or “gentle”. In seeking to match these labels, women invest substantial time in refining their posts, which not only reinforces objectification but also consumes emotional energy and time, while further limiting the expression of individuality, intellect, and inner worth.

#### Diverse aesthetics under counter-disciplinary practices

The “imagination tax” is not merely about surface-level embellishment; it is also closely tied to the pursuit of social recognition. Content shared online can be deliberately curated and edited. This grants individuals greater control over their public image. As a result, people often modify their self-presentation based on audience feedback, continuously aligning themselves with “social expectations” and thereby “performing the self”. This gives rise to “imagined consumption”—a behavior in which individuals construct their identity and social status through the consumption of specific products, services or visual representations.

The “imagination tax” is, however, no longer a one-way imposition but is increasingly subject to reflection and reduction. The decentralization of media platforms allows individuals to present unique aspects of themselves and break away from traditional aesthetic constraints. Whether through unconventional fashion choices or avant-garde makeup styles, such expressions have the potential to gain popularity rapidly, thus fostering the dissemination of personalized aesthetic values.

Another interviewee, Maomao (29, contract staff at a government agency), stated that for those who passively scroll through social media without awareness, the content they are exposed to tends to become increasingly conservative. However, in her view, if one is conscious and seeks greater bodily and mental freedom, one can intentionally search for more diverse aesthetic styles while browsing social media. Today's social media platforms are becoming more advanced and are evolving toward greater individualization, as Chen Collins (22, law student) observed:

I really enjoy experimenting with different styles. My skin is relatively dark, and certain clothes make me appear even darker. But if I perceive darkness as a problem, then I am already subjected to disciplinary aesthetic standards. By trying out different styles, I actually enrich that standard, rather than remain enslaved by it. (Interview, Chen Collins)

Mainstream aesthetic standards tend to receive heightened commercial attention in the short term. However, as more users engage in critical reflection and active resistance, individualized and diversified aesthetics are gradually gaining broader recognition and appreciation. When confronted with prevailing trends, Chinese young women are increasingly incorporating their own unique cultural backgrounds and emotional perspectives. Sometimes they even challenge traditional aesthetic norms. In doing so, they contribute to the diversification of styles and engage in a “counter-discipline” against singular aesthetic standards. Social media platforms can provide ample space for aesthetic freedom that enables individuals to discover and express their unique identities within a globalized context, thereby facilitating the expansion of aesthetic pluralism.

## Discussion

In an era of rapid digital media development, the self-objectification exhibited by contemporary Chinese young women when constructing their aesthetic cognition has evolved beyond an individual psychological phenomenon and has increasingly become a shared social mentality. This reflects the way Chinese young women perceive themselves within a broader sociocultural context and highlights the influence of media environments on the construction of female identity. It also reveals the underlying mechanisms through which public discourse shapes the social value assigned to women's identities.

Objectification functions as an intermediary mechanism on social media platforms. Factors such as algorithmic recommendations, interactive mechanisms and usage frequency significantly influence women's perception and expression of their self-image. Although these platforms offer a space for self-representation, they also reinforce mainstream aesthetic norms, thereby intensifying women's self-surveillance and anxiety. By positioning themselves as objects to be viewed, women risk outsourcing their sense of self-worth to external evaluations. In managing and curating their self-image online, women are not merely shaping a personal identity but are also engaging in the broader negotiation of sociocultural value systems.

This type of impression management is shaped by the structural logic of platforms, algorithmic targeting and patterns of user interaction. Social media is thus both a stage for aesthetic expression and a powerful agent for constructing aesthetic norms. Within the tension between individual expression and social standardization, women's aesthetic values face increasing challenges. A healthy sense of self should originate from inner strength and reliable capabilities, not from the voices of external judgement. The realization of female self-worth should not be contingent upon others' validation. Social media platforms, therefore, bear some responsibility in fostering a healthier and more inclusive aesthetic environment.

The algorithmic design of digital platforms has, to some extent, restricted aesthetic diversity. Platforms should therefore improve the structures of their algorithms to reduce the promotion of homogeneous aesthetic preferences and encourage the dissemination of more diverse and personalized content, thus avoiding the reinforcement of fixed appearance norms. For example, platforms could consider hiding the “like” function or minimizing the visual emphasis on external attributes such as appearance or body shape, as this would alleviate users' excessive attention to such features and reduce the limitations social media imposes on aesthetic cognition. Platforms could also strengthen users' media literacy by incorporating educational content to help them identify manipulated images and unhealthy beauty ideals (Liu, 2023, p. 103–110). Dedicated educational sections or campaigns against appearance-related anxiety—such as a “No Filter Challenge” or a “Day of Authentic Sharing” on Little Red Book—could enhance users' self-recognition and body acceptance.

Chinese young women should strive to transcend the aesthetic frameworks delineated by platform algorithms and technologies. Instead, they should construct self-awareness through self-pleasing and authentic representation. Rational discernment in the face of new media trends is essential for achieving a harmonious aesthetic paradigm that values both individual beauty and collective diversity. Women could also avoid an excessive reliance on external evaluations to define their self-worth by improving media literacy, learning to critically evaluate commercialized content on social media and fostering critical thinking. This transition—from being passively objectified under the “gaze” to actively “seeing” one's inner self—would mark a shift from being looked at to truly seeing oneself. Recognizing that self-worth is fundamentally rooted in one's inner individuality and capability, women can learn to use social media judiciously, focus on genuine personal development and ultimately achieve a more complete and confident sense of identity.

## Data Availability

The raw data supporting the conclusions of this article will be made available by the authors, without undue reservation.
